# Comparison between 5-aminolevulinic acid photodynamic diagnosis and narrow-band imaging for bladder cancer detection

**DOI:** 10.1186/s12894-021-00946-w

**Published:** 2021-12-22

**Authors:** Hiroki Hagimoto, Noriyuki Makita, Yuta Mine, Hidetoshi Kokubun, Shiori Murata, Yohei Abe, Masashi Kubota, Naofumi Tsutsumi, Toshinari Yamasaki, Mutsushi Kawakita

**Affiliations:** Department of Urology, Kobe City Medical Center General Hospital, 2-1-1 Minatojima-Minamimachi, Chuo-ku, Kobe, Hyogo 650-0047 Japan

**Keywords:** Bladder cancer, 5-aminolevulinic acid, Narrow-band imaging, Photodynamic diagnosis, Transurethral resection of bladder tumour

## Abstract

**Background:**

To compare 5-aminolevulinic acid (5-ALA)-mediated photodynamic diagnosis (PDD) with narrow-band imaging (NBI) for cancer detection during transurethral resection of bladder tumour (TURBT).

**Methods:**

Between June 2018 and October 2020, 114 patients and 282 lesions were included in the analysis. Patients were orally administered 5-ALA (20 mg/kg) 2 h before TURBT. The bladder was inspected with white light (WL), PDD, and NBI for each patient, and all areas positive by at least one method were resected or biopsied. The imaging data were then compared to the pathology results.

**Results:**

The sensitivities of WL, PDD, and NBI for detecting urothelial carcinoma were 88.1%, 89.6%, and 76.2%, respectively. The specificity, positive predictive value, and negative predictive value for detecting urothelial carcinoma were 47.5%, 80.9%, and 61.3%, respectively, for WL; 22.5%, 74.5%, and 46.2%, respectively, for PDD; and 46.3%, 78.2%, and 43.5%, respectively, for NBI. PDD was significantly more sensitive than NBI for all lesions (*p* < 0.001) and carcinoma in situ (CIS) lesions (94.6% vs. 54.1%, *p* < 0.001).

**Conclusions:**

PDD can increase the detection rate of bladder cancer, compared to NBI, by greater than 10%. Therefore, 100% of CIS lesions can be detected by adding PDD to WL.

## Introduction

Bladder cancer is the ninth most frequently diagnosed malignancy in the world [1] and is the fourth leading cause of death among men [2]. The 5-year recurrence rate of non-muscle-invasive bladder cancer ranges from 50 to 70%, and the reported 5-year progression rate ranges from 10 to 30% [3]. Transurethral resection of bladder tumour (TURBT) with intraoperative detection of the cancer by white light (WL) is the standard treatment for non-muscle invasive bladder cancer [4]. However, it is difficult to detect flat lesions, including carcinoma in situ (CIS), with WL alone, and the high rate of intravesical recurrence of CIS is problematic. Therefore, it is important to accurately detect CIS in order to be able to eliminate any residual tumour, and more importantly to identify the patients who require further treatment such as intravesical BCG. It is known that the addition of photodynamic diagnosis (PDD) and narrow-band imaging (NBI) to WL increases the detection rate of cancer in flat lesions that do not appear as distinct lesions with conventional WL [5–8]. They also are associated with lower recurrence rates compared to WL only [2, 9–11]. PDD is a technique that exploits the property of tumours or rapidly proliferating cells to emit red fluorescence during cystoscopy using blue light, after oral or intravesical administration of a photosensitizing precursor such as 5-aminolevulinic acid (5-ALA). NBI is a technique that utilises the fact that tissue penetration by light depends on its wavelength. By exposing the bladder wall to light with two narrow-banded wavelengths, that are easily absorbed by haemoglobin, capillaries on the surface of the mucosa are displayed as brown and blood vessels inside the submucosa as blue-green, highlighting the tumour. PDD using 5-ALA (5-ALA-PDD) for non-muscle invasive bladder cancer reduces the risk of recurrence [12], and has a real-world sensitivity and specificity of 90.1% and 61.2%, respectively [13]. For NBI, a meta-analysis showed that the pooled sensitivity and specificity for non-muscle invasive bladder cancer were 94.8% and 65.6%, respectively [14].

The 2021 European Association of Urology (EAU) guidelines strongly recommend taking biopsies from both abnormal-looking urothelium and normal-looking mucosa (mapping biopsies from the trigone, bladder dome, right, left, anterior, and posterior bladder wall) when cytology is positive, in case of a history of high grade (G3) tumours, and for tumours with non-papillary appearance. Moreover, PDD-guided biopsies should be used if equipment is available. However, bladder biopsy using NBI during TURB is weakly recommended [15].

Some studies have examined the usefulness of combining PDD and NBI for the detection of flat lesions [16, 17]. However, no comparative studies exist between 5-ALA-PDD and NBI for the detection of urothelial carcinoma, including both protruded and flat lesions. The purpose of this study was to compare 5-ALA-PDD and NBI in terms of bladder cancer detection capability in the same patients.

## Material and methods

### Study cohort and design

**Fig. 1:**
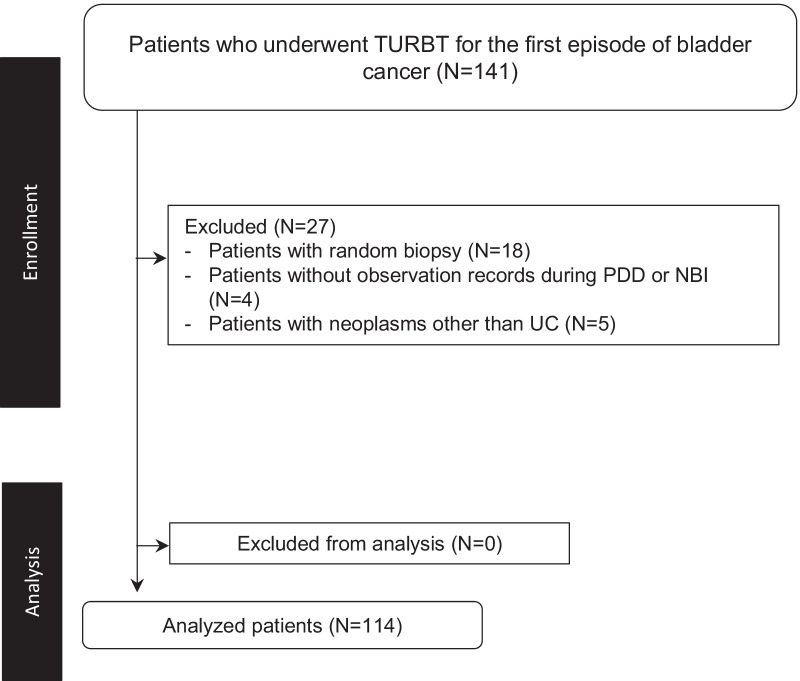
Flow diagram of patient enrolment

This single-centre retrospective cohort study was approved by the institutional review board (IRB No. zn210416) and conformed to the provisions of the Declaration of Helsinki (as revised in Fortaleza, Brazil, October 2013). All patients were informed of the efficacy and adverse events associated with 5-ALA-PDD and NBI, and we obtained informed consent from all patients before the operation. A total of 141 patients with primary bladder cancer, who underwent 5-ALA-PDD and NBI-assisted TURBT between June 2018 and October 2020, were included in the study. Eighteen patients with random bladder biopsies, five patients with histology consistent with prostate cancer, squamous cell carcinoma, or renal cell carcinoma, and four patients with no observation records for either NBI or 5-ALA-PDD were excluded. Included in the analysis were 114 patients with 282 lesions. Figure 1 shows the flow diagram of the study. We recorded the data on the surgeons' interpretation of the images prospectively, and analysed them retrospectively.

### Surgical procedure

**Fig. 2:**
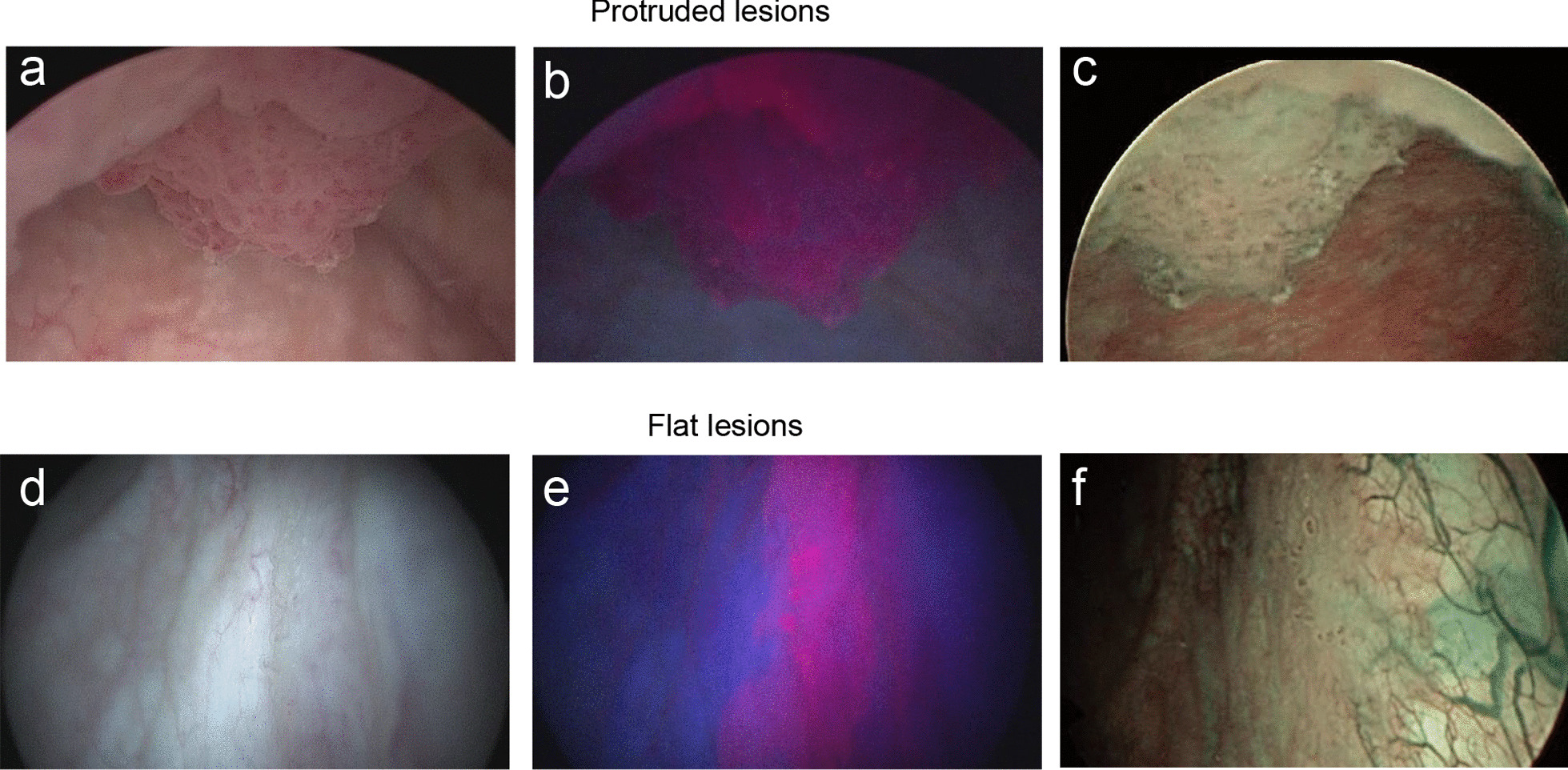
Representative endoscopy images of positive tumour findings in protruded and flat lesions using **a** WL, **b** PDD, **c** NBI

All patients were administered 20 mg/kg of 5-ALA 2 h before the start of surgery. In all cases, the positive and negative lesions were determined by two urologists. At the time of TURBT, the bladder was inspected with WL, 5-ALA-PDD, and NBI, and all lesions deemed positive by at least one modality were resected. WL was used first and was followed by either 5-ALA-PDD or NBI depending on the surgeon’s preference. We used AUTOCON® III 400 (KARL STORZ, Tuttlingen) for PDD and OES ELITE 30° optic tube Φ4 mm (Olympus, Tokyo) for NBI. Bladder inspection with each modality occurred consecutively. Figure 2 shows a typical view by each modality. After TURBT, 50 mg of epirubicin hydrochloride in 50 mL of saline solution was instilled into the bladder through a Foley catheter, which was clamped for 30 min. The patients were kept out of direct sunlight for 48 h after surgery. We reviewed the pathology results and the intraoperative detection frequencies of each modality, and, accordingly, determined the cancer detection rate and sensitivity for each modality. Grading was performed according to the 2016 World Health Organization classification.

### Safety assessment

We noted the incidence of side effects common with 5-ALA, such as vomiting, liver damage, and hypotension. Grades were determined based on the Common Terminology Criteria for Adverse Events, version 6.

### Statistical analysis

The extracted data from the prospective database included age, sex, body mass index, preoperative urine cytology, operation time, surgeon, anaesthetic method, pathological examination, length of hospital stay, readmission, in-hospital complications, and Clavien-Dindo grade [18]. We calculated and compared the sensitivity, specificity, positive predictive value, and negative predictive value for each lesion. Significant differences between the two groups were assessed based on McNemar's odds ratio using standard analysis software (BellCurve for Excel; Social Survey Research Information Co., Ltd.). All statistical tests were two-tailed, with *p* < 0.05 indicating statistical significance.

## Results

In total, 282 lesions from 114 patients were included in the study, and all of them were available for data analysis. A total of 24 patients were observed with PDD first followed by NBI, and 90 patients were observed with NBI first followed by PDD. Table 1 shows the patients’ characteristics. The specimens’ histology is presented in Table 2. Urothelial carcinoma (UC) was diagnosed in 202 specimens, whereas the remaining 80 were benign. Of the benign specimens, 3 were consistent with dysplasia, 13 with papilloma, and the rest were normal tissues. Table 3 shows the sensitivity of each modality for the detection of UC and the specificity for benign lesions. As shown, 5-ALA-PDD had a higher overall sensitivity than NBI (89.6% vs. 76.2%, *p* < 0.001). It also was more sensitive than NBI for CIS lesions (94.6% vs. 54.1%, *p* < 0.001). We were able to detect 100% of the CIS lesions with combined WL + PDD (WP), thus increasing the rate of detection by 35.1% compared with that of WL alone.

**Table 1:** Patients’ background and tumour characteristics

Enrolled patients	Total (n = 114)
Age, years (median)	76 (IQR: 60–81)
Gender	
Female, n(%)	35 (30.7%)
Male, n(%)	79 (69.3%)
Body weight, kg, median(IQR)	53 (45.3–62.8)
Operation time, min, median (IQR)	60 (38–79)
Preoperative cytology	
Positive, n(%)	25 (21.9%)
Negative, n(%)	85 (74.6%)
N/A, n(%)	4 (3.5%)
Anesthetic method	
General, n(%)	47(41.2%)
Lumbar, n(%)	67 (58.8%)
5-ALA exposure time, min, median(IQR)	177(157.5–207)
Number of lesions	282
Protruded papillary, n(%)	189 (67.0%)
Protruded non-papillary, n(%)	17 (6.0%)
Flat lesions, n(%)	76 (27.0%)
Number of tumors per person, median(IQR)	2 (2–3)

5-ALA: 5-aminolevulinic acid, IQR: interquartile range

**Table 2:** Specimen histology

Tumors	N (%)
Total	282
UC	202 (71.6)
Stage	
Ta	125 (44.3)
T1	26 (9.2)
T2	14 (5.0)
CIS	37 (13.1)
Grade	
Low	16 (5.7)
High	186 (66.0)
Benign	80 (28.4)
Dysplasia	3 (1.1)
Papilloma	13 (4.6)
Normal	64 (22.7)

CIS: carcinoma in situ; UC: urothelial carcinoma

**Table 3:** Sensitivity of each modality for detection of UC and specificity for benign lesions

	WL (%)	NBI (%)	PDD (%)	WN (%)	WP (%)	WNP (%)
Sensitivity						
All UC	178/202 (88.1)	154/202(76.2)	181/202 (89.6)	183/202 (90.6)	201/202 (99.5)	202/202 (100)
CIS	24/37 (64.9)	20/37 (54.1)	35/37 (94.6)	26/37 (70.3)	37/37 (100)	37/37 (100)
Non-CIS	153/165 (92.7)	134/165 (81.2)	145/165 (87.9)	156/165 (94.5)	162/165 (98.2)	163/165 (98.8)

	WL (%)	NBI (%)	*PDD* (%)
Specificity			
All benign	38/80 (47.5)	37/80 (46.3)	18/80 (22.5)

CIS, carcinoma in situ; NBI, narrow-band imaging; PDD, photodynamic diagnosis; UC, urothelial carcinoma; WL, white light; WN, white light with NBI; WNP, white light with NBI and PDD; WP, WL + PDD

Adverse events, including hypotension (N = 61), elevated liver enzyme levels (N = 4), and vomiting (N = 20) were less than grade 3. All incidents (N = 61) of hypotension were of grade 2 or lower according to Common Terminology Criteria for Adverse Events version 5.0. Of the 67 patients who received lumbar anaesthesia, 22 patients (32.8%) received intraoperative vasopressors, and of the 47 patients who received general anaesthesia, 39 patients (83.0%) received vasopressors during induction. None of the patients required vasopressors after the surgery. Elevated levels of aspartate aminotransferase were found in four patients, one of whom was classified as grade 2, and three were classified as grade 1. All the patients who had vomiting were classified as grade 1. They had vomiting postoperatively within a median of 5.9 (interquartile range: 1.3–7.4) hours following 5-ALA administration. In all cases, nausea had disappeared by the next day. Regarding postoperative complications, there were 12 cases of grade 1 or higher as per the Clavien-Dindo classification. There was only one case of grade 3a, which required ERCP for fever due to postoperative recurrence of hepatocellular carcinoma. The remaining 11 cases were grade 2 or lower, including temporary postoperative urinary retention and clot retention requiring manual bladder washing to eliminate the blood clot.

## Discussion

In this study, 5-ALA-PDD was superior and considered more useful than NBI in reducing the number of missed tumours. The detection rate of pTis lesions in the WL + PDD group was 100%, and the rate of additionally detected CIS with WL + PDD was increased by 35.1% compared with WL alone. Although this 100% sensitivity is partially due to the fact that we did not sample negative lesions for PDD, NBI, or WL; thus, it is possible that some patients may have had an undetected CIS that we were not aware of; 5-ALA-PDD proved to be particularly useful in detecting flat lesions such as CIS. The present study determined a lower specificity for PDD than other reports [12, 13]. This may be due to the lack of a systematic biopsy of the negative areas and the exclusion of cases for whom random biopsies were performed, such as cold-cup biopsies of normal appearing lesions, or biopsies on lesions that were even slightly positive for PDD to avoid missing cancer. This is one of the limiting factors that prevented us from examining the specificity in the present study as it was a retrospective study.

According to the literature, transient elevation of liver enzymes of grade 3 or lower, as well as hypotension, can occur due to 5-ALA, and caution is recommended with its use [19, 20]. In this study, there was only one case of grade 3a adverse effects, which was not associated with 5-ALA. Moreover, the rest of the adverse events were grade 2 or lower, and could be easily managed.

The present study has several limitations which are as follows: (1) the small number of patients included; (2) single-centre retrospective design; (3) inter-observer and intra-observer bias, which was due to the subjective nature of visual estimation and could be potentially be addressed by the development of methods to quantify visual information; and (4) procedure bias introduced by the order of use of each modality and the time between 5-ALA administration and bladder inspection in PDD. This study includes the possibility that the sensitivity of PDD was increased because of the bias in which many of the patients were observed first with NBI followed by PDD. There is a phenomenon called photobleaching, in which 5-ALA degrades upon repeated illumination and the detection rate is reduced [21]. Although observation at 2–4 h after 5-ALA oral administration is recommended, the time of 5-ALA exposure to light may be less important, as it has been shown that no significant difference exists between exposure times of 2–3 h and 4 h or more [13]. Recent studies have shown a fluorescence enhancement effect using polyethylene glycol-modified titanium dioxide nanoparticles [21], which may be useful for reducing the bias caused by photobleaching.

## Conclusions

5-ALA-PDD at the time of TURBT increased the detection rate of bladder cancer and especially of flat lesions such as CIS, compared to NBI. Furthermore, the combination of PDD with WL, which is the current standard of care, achieves 100% sensitivity in the detection of CIS. Prospective studies will be needed to verify these results.

## Data Availability

The datasets used and/or analysed during the current study are available from the corresponding author on reasonable request.

## Abbreviations

5-ALA: 5-Aminolevulinic acid
CIS: Carcinoma in situ
NBI: Narrow-band imaging
PDD: Photodynamic diagnosis
TURBT: Transurethral resection of bladder tumour
WL: White light
