# Correction to: Comparison between 5-aminolevulinic acid photodynamic diagnosis and narrow-band imaging for bladder cancer detection

**DOI:** 10.1186/s12894-022-00970-4

**Published:** 2022-02-17

**Authors:** Hiroki Hagimoto, Noriyuki Makita, Yuta Mine, Hidetoshi Kokubun, Shiori Murata, Yohei Abe, Masashi Kubota, Naofumi Tsutsumi, Toshinari Yamasaki, Mutsushi Kawakita

**Affiliations:** Department of Urology, Kobe City Medical Center General Hospital, 2-1-1 Minatojima-Minamimachi, Chuo-ku, Kobe, Hyogo 650-0047 Japan

## Correction to: BMC Urol 2021 21(1):180 10.1186/s12894-021-00946-w

Following publication of the original article [1], it was noted that due to a typesetting error the Fig. 1 was incorrect. The correct figure is given below.

**Fig. 1:**
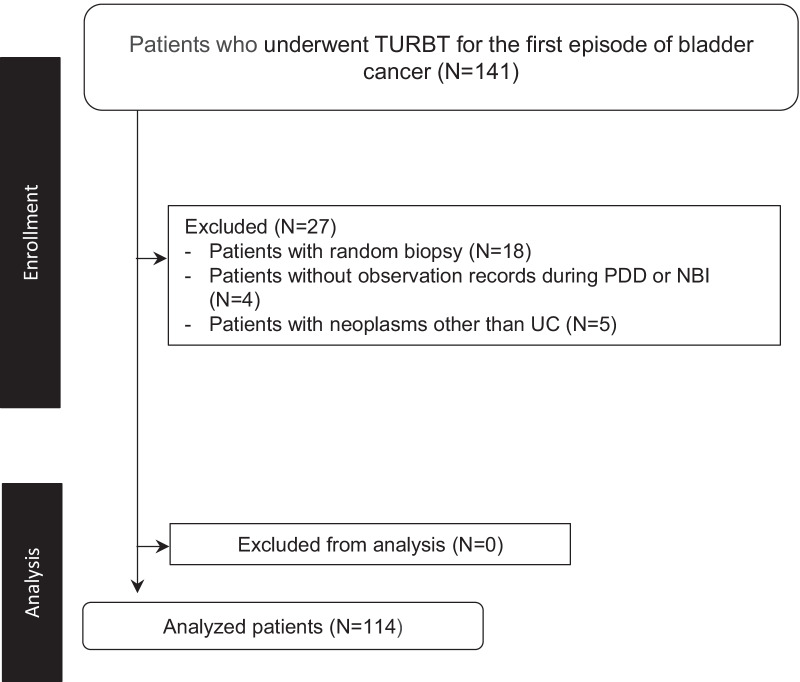
Flow diagram of patient enrolment

Second, the authors would like to correct the reference number in the second paragraph under the heading Discussion section.

The sentence should read:

Although observation at 2–4 h after 5-ALA oral administration is recommended, the time of 5-ALA exposure to light may be less important, as it has been shown that no significant difference exists between exposure times of 2–3 h and 4 h or more [13].

The original article [1] has been corrected.
